# Accuracy of the Different Materials Used to Fabricate a Verification Jig of Implant-Supported Fixed Complete Dental Prostheses: An In Vitro Study

**DOI:** 10.7759/cureus.29794

**Published:** 2022-09-30

**Authors:** Mawaddah S Aljohani, Heba A Bukhari, Mayar Alshehri, Ahmed Alamoudi

**Affiliations:** 1 General Dentistry, Faculty of Dentistry, King Abdulaziz University, Jeddah, SAU; 2 Oral Biology, Faculty of Dentistry, King Abdulaziz Univeristy, Jeddah, SAU

**Keywords:** implant passive fit, framework fit, biological and mechanical complication, 3d assessment, implant framework, implant master cast, verification jig

## Abstract

Introduction

The passive fit of a full arch implant-supported prosthesis is one of the elements influencing implant success. Achieving the passive fit of a prosthesis requires verification of the master cast before the fabrication of the framework. A verification jig is a common way to verify the implant master cast and ensure the accuracy of the implant impression and produced cast. Different materials can be used to fabricate verification jigs, and each material exhibits different dimensional changes. In this study, we compared the accuracies of verification jig materials by 3D assessment.

Materials and methods

A type IV stone cast with four implant analogs was constructed and used as a control. Verification jigs were constructed from five different materials, and test casts were made from these jigs and poured using low expansion stone (type IV), resulting in five groups (n=5). All test casts and the control cast were scanned using a lab scanner. The scans of test casts were superimposed on that of the control cast for 3D accuracy assessment. The distortion of the implant analogs was recorded using Geomagic Design X and Geomagic Control X software (3D Systems, Rock Hill, South Carolina, USA). Statistical differences in the 3D accuracies between the five groups were analyzed using the Kruskal-Wallis test.

Result

The photopolymerizable resin group had a mean value of 23.16 (± 0.88) µm; the composite group had a mean value of 46.72 (± 2.122) µm; the GC pattern group had a mean of 23.51 (± 0.736) µm; the type III stone group had a mean of 19.84 (± 1.017) µm; the type IV stone group had a mean of 18.72 (± 0.819) µm. The Kruskal-Wallis test indicated that there were statistically significant differences between groups 2 (composite), 4 (type III stone), and 5 (type IV stone).

Conclusion

The most accurate cast was produced by type IV stone, followed by type III stone, photopolymerizable resin, GC pattern, and composite in order of decreasing accuracy. Within the limitations of the study, a material with low distortion and high accuracy is recommended when fabricating verification jigs of implant-supported complete dental prostheses.

## Introduction

Full-mouth rehabilitation with implant-supported fixed complete dental prostheses has become a routine procedure that has gained popularity among clinicians [1]. The framework of prostheses must fit precisely and passively on dental implants to prevent unfavorable stresses on the implant components and surrounding bone [2]. Any degree of prostheses misfit will lead to mechanical and biological complications [3-5]. Mechanical problems include screw-loosening of the prosthesis and its abutments, fracture of the framework, and chipping of the veneer material [5-7]. Biological consequences of a misfit include unfavorable tissue reactions, tissue soreness, bone loss, pain, and loss of osseointegration [5]. Therefore, an accurate impression is required to fabricate an accurate master cast and passive framework [8].

There is no clear definition of acceptable passive fit. It was defined by several authors hypothetically: Branemark proposed that passive fit at a level of 10 μm is acceptable [9]. Jemt considered a 150-μm gap or less between the abutment and prosthesis an acceptable passive fit of an implant framework [10]. Karl et al. described passive fit as the absence of strain on the implant when the framework is in place [11]. Patterson defined a passive fit as when there is no gap between the abutment and framework and no strain or stress after screw-torquing [12].

There are several clinical methods of evaluating implant-framework fit, such as alternating finger pressure, radiographs, the one-screw test, the screw-resistance test, direct visual inspection, disclosing media, and tactile sensation; however, there is no reliable standard test to assess passive fit [8]. One common technique to assess the accuracy of a produced cast and ensure the accuracy of the implant impression prior to framework fabrication is the verification jig [13-15]. In addition, verification jigs can be used if the master cast is not accurate by correcting the position of the implant analogs in the cast [13-15]. Verification jigs can be fabricated from different materials, such as light-polymerizing acrylic resin, auto-polymerizing acrylic resin, composite resin, and dental stone [13,16,17]. Each material has different dimensional accuracy, which can affect the accuracy of the jigs and result in an inaccurate cast and framework [18].

Photopolymerizable resin is a light-curing acrylic resin material. It is used mainly for fabricating custom impression trays, base plates, and bite blocks in prosthodontics treatment. It is composed of acrylates/methacrylates and glass with no monomer causing less polymerization shrinkage [19]. Composite resin has been used as a restorative material in modern dentistry and has several clinical applications with numerous desirable qualities such as esthetic appearance, and adequate physical and mechanical properties. One of the limitations of these white-colored materials is the polymerization shrinkage which ranges from 1.35% to 7.1% during curing and can be reduced by incremental technique [20]. Pattern resin is a self-curing acrylic resin that is used by dentists and laboratory technicians for making copings, custom-made post and core, bars, prosthetic attachments, clasps, telescopic crowns, as well as in some soldering techniques. It sets quickly and can be modulated easily and is the ideal material for dentists and laboratories. As an acrylic resin GC pattern resin has some polymerization shrinkage and 80% of the shrinkage occurs within 17 minutes, insignificant changes happen after 24 hours [18]. Gypsum is a commonly used material in cast and die fabrication. There are five types of gypsum products - type I: plaster impression; type II: plaster model; type III: dental stone; type IV: high strength, low expansion dental stone; type V: high strength, high expansion dental stone. Gypsum products have remarkable properties such as high strength, high compressive strength, hardness, and minimal expansion. One drawback of gypsum material is that it displays some linear expansion after setting and leads to dimensional changes varying from 0.08% to 0.3% [21].

Papaspyridakos et al. compared three verification jig materials: GC pattern resin, Fixpeed resin, and Triad gel. They found no significant differences between these materials when tissue-level implants were parallel [16]. Other materials have not been investigated, and there is a lack of data about which material is best for constructing verification jigs. Therefore, the purpose of this in vitro study was to compare the accuracy of different materials used to fabricate verification jigs by comparing the generated fabricated casts.

## Materials and methods

**Figure 1:**
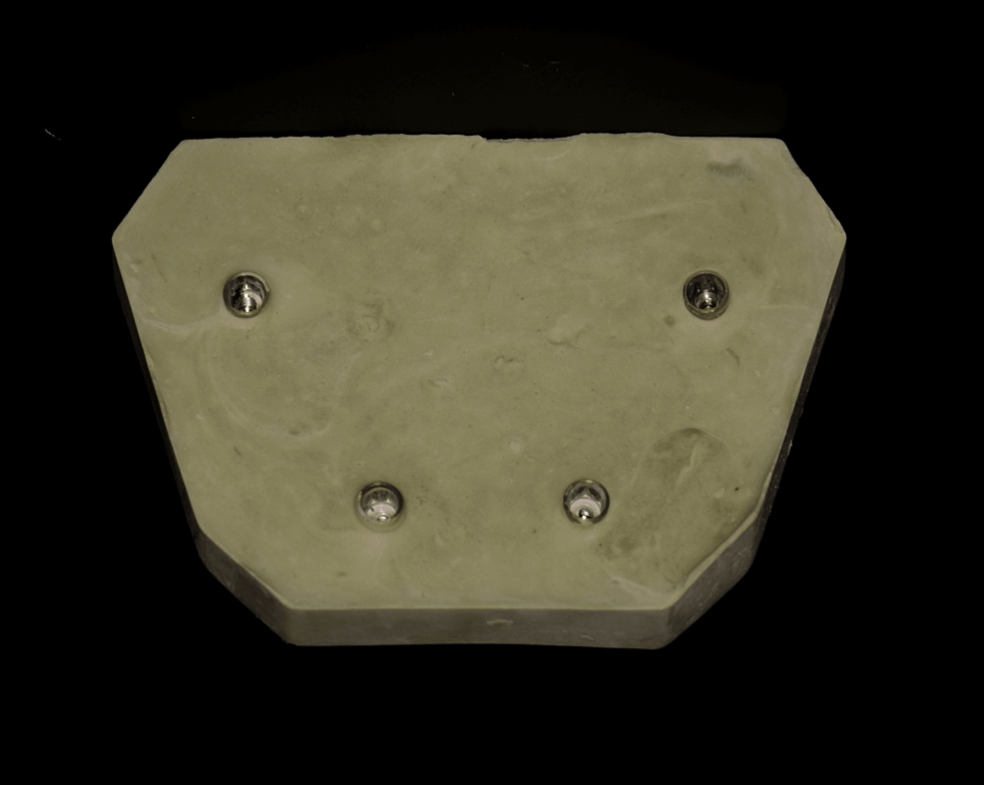
Control cast with four internal connection implant analogs

A stone dental cast with four analogs of internal implant connections (bone-level conical connection; Nobel Biocare, Zurich, Switzerland) was constructed using type IV stone (extra-hard natural stone; Protechno, Girona, Spain), which represented the edentulous mandible and served as a control (Figure 1). Two implant analogs were placed anteriorly, and two implant analogs were placed posteriorly, resembling a clinical scenario. The two implants in the anterior region were parallel to each other, as well as the two implants in the posterior region.

Verification jigs using non-engaging temporary abutments (Nobel Biocare) and five different materials were created on the control stone model to fabricate five stone casts from each group with a total of 25 casts. Each verification jig material was grouped as follows.

Group 1

**Figure 2:**
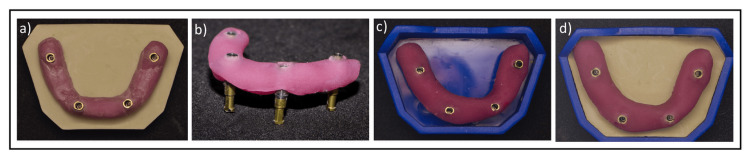
Steps of test cast fabrication produced from photopolymerizable resin verification jig a) Temporary abutments were tightened to the implant analogs on the control cast b) Attached temporary abutments to verification jig after they were untightened from the implant analogs of the control cast and connected to another implant analogs c) Connected temporary abutments to implant analogs were placed in a silicone mould d) Low-expansion type IV stone was poured to construct the test cast. This method was used to construct all test casts from the different materials used in this study.

**Figure 3:**
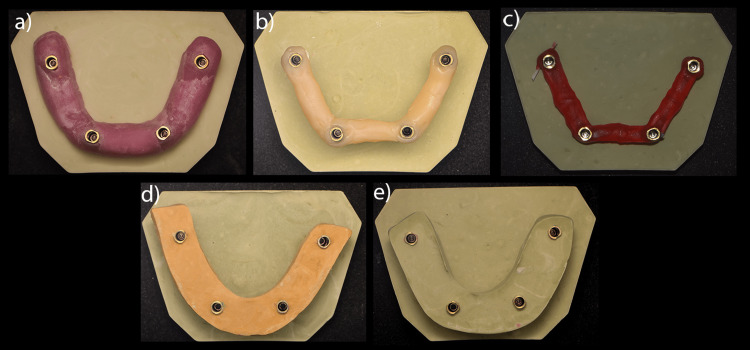
Representative casts with verification jigs made of a) photopolymerizable resin, b) composite, c) GC pattern, d) stone type III and e) stone type IV.

Stone cast fabricated using photopolymerizable resin verification jig (n=5): Four non-engaging temporary abutments were tightened on the implant analogs of the control cast and splinted together using photopolymerizable resin (ECO-TRAY LUZ, Protechno) creating five verification jigs (Figure 2a and Figure 3a). Once the photopolymerizable resin jig was created, it was cured for three minutes using a Triad 2000 VLC light curing unit (Dentsply Sirona, Charlotte, North Carolina, USA). The jig was then sectioned into three segments, and a small amount of material was used to fill the gaps to compensate for polymerization shrinkage, before being cured again for an additional three minutes. To construct the test cast, the jig was unscrewed and removed from the cast. The temporary abutments were then connected to the implant analogs (Figure 2b), and placed in a silicone mold to create the test cast (Figure 2c). The test casts were poured with low expansion type IV stone (extra-hard natural stone, Protechno) (Figure 2d). After the stone was set, the temporary abutments were untightened from the cast and were ready for 3D assessment. This method was performed five times to produce five test casts.

Group 2: Stone cast fabricated using composite verification jig (n=5): Five verification jigs were prepared using light-cured nanohybrid composite resin (DiaFil, Guidonia, Rome, Italy) using the same steps as described above to fabricate the test casts. The curing light (Light Curing Unit iLED Plus; Guilin Woodpecker Medical Instrument Co. Ltd., Guangxi, China) was held at a distance of 1 cm distance, and each side of the jig was cured for 20 seconds for a total of 60 seconds (Figure 3b).

Group 3: Stone cast fabricated using GC pattern verification jig (n=5): Five verification jigs were prepared using auto-polymerizing resin (GC pattern resin). The jigs were left for 24 hours to allow complete polymerization. Then the jig was sectioned, and resin was used to fill the gaps between the separated segments (Figure 3c). The non-engaging temporary abutments were untightened after 15 minutes to make the test casts following the same procedures described above. 

**Figure 4:**
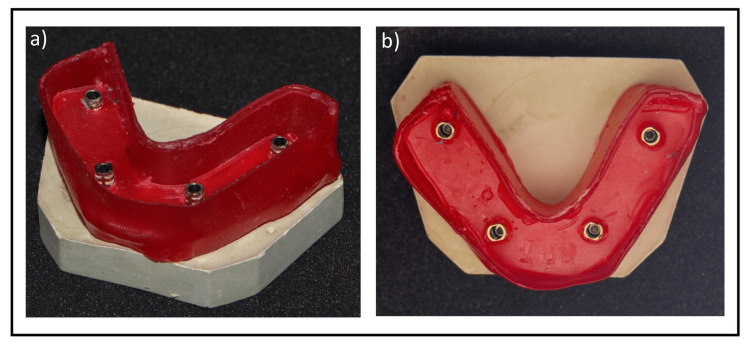
Wax mould was created and used to hold the stone a) Angled view, b) Top view of wax mould

Group 4: Stone cast fabricated using type III dental stone verification jig (n=5): Five verification jigs were prepared using type III dental stone (hard natural stone, Protechno) (Figure 3d). The water:powder ratio used was recommended by the manufacturer. A mould made of boxing wax (Coltene, Altstätten, Switzerland) was used to hold the stone (Figure 4). The same procedures described above were used to fabricate the test casts. 

Group 5: Stone cast fabricated using type IV dental stone verification jig (n=5): Five verification jigs were prepared using high strength, low expansion (extra-hard natural stone, Protechno) type IV stone (Figure 3e). The water:powder ratio used was recommended by the manufacturer. A mould of boxing wax (Coltene) was used to hold the stone (Figure 4). After the stone had set, the same procedures described above were followed to fabricate the test casts.

Three-dimensional (3D) accuracy

**Figure 5:**
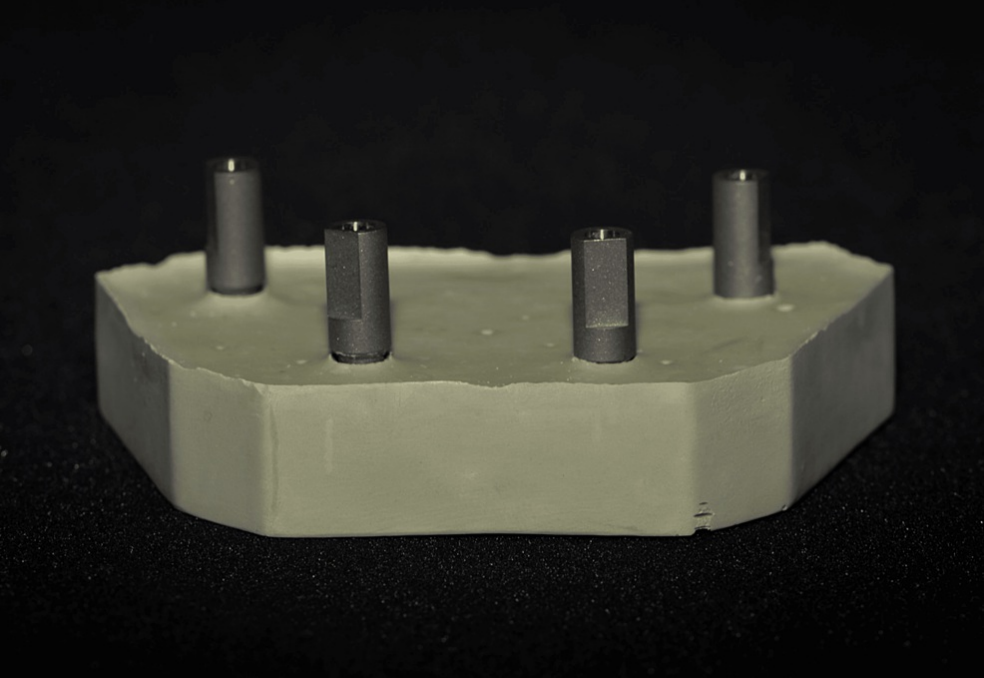
A control cast with four scan bodies (Nobel Biocare) on four implant analogs for the 3D digital scan. The same scan bodies were placed on the same implant analogs in each test cast.

Four scan bodies (Nobel Biocare Nobelactive Scanbody (Ti) RP 4.3, Smart Implant Solution, Nobel Biocare, Kloten, Switzerland) were placed on the four implant analogs for assessment of their 3D accuracy (Figure 5). A 3D scanner (KaVo ARCTICA Autoscan, KaVo Dental, Berlin, Germany) was utilized to capture the 3D orientation and position of the implants. This scanner uses very sensitive 3D sensors in conjunction with striped light projection to gather detailed images and convert them into a 3D model. The scanner does not require powder. All the scanning was performed by one operator. The scan of the control cast was used as the reference. The same scan bodies were placed on the same implant analogs in each cast for all 3D digital scans. Following scanning of the casts of the test groups and the control cast, Standard Tessellation Language (STL) files were obtained. Each scan of the test groups was superimposed over the control scan. Geomagic Design X and Geomagic Control X software (3D Systems, Rock Hill, South Carolina, USA) were used to measure 3D differences between the control STL file and the 25 test STL files. 

Statistical analysis

Descriptive statistics were calculated for the five groups in this study (photopolymerizable resin, composite, GC pattern, type III stone, and type IV stone). A test for normality was done for the data of this study and it showed uneven distribution. The Kruskal-Wallis test was used to analyze the differences in 3D accuracy between the five groups and the control. The result was considered statistically significant if p<0.001. 

## Results

**Figure 6:**
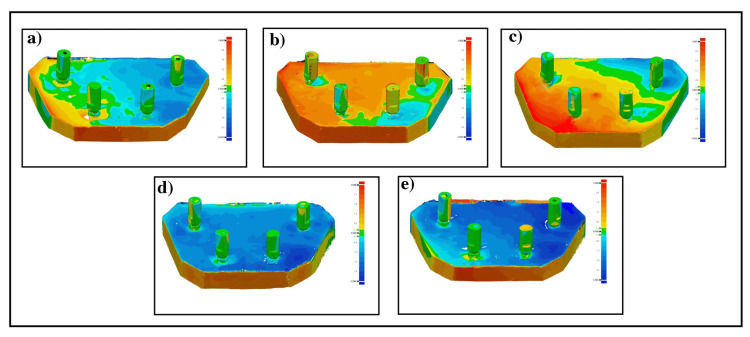
Colorimetric maps of superimposition of control scan and test scans of (a) photopolymerizable resin group, (b) composite group, (c) GC pattern group, (d) type III stone group, and (e) type IV stone group.

A 3D assessment was performed after superposition of the control cast scan and test cast scans using Geomagic Design X and Geomagic control software as presented as seen in Figure 6. Color gradient-map of superimposition of control cast and photopolymerizable resin casts scans showed green color with few areas of yellow and blue colors over the implant scan bodies indicating minor deviations (Figure 6a). Colorimetric map of the control cast and composite group scans displayed yellow, blue, and red colors more than green indicating a significant deviation between the position of the implants in the control cast and produced casts from the composite verification jigs (Figure 6b). The superimposition of control cast and GC pattern group scans color-gradient map showed mainly green color with few surface areas of blue and yellow colors suggesting minimal 3D implant deviation (Figure 6c). Colorimetric map of control cast scan and type III and IV scans revealed primarily green color with few areas of blue and yellow colors over the implant scan bodies indicating high accuracy with minimum implant deviation (Figure 6d, 6e). 

The mean ± SD, minimum and maximum values of the 3D deviations are shown in Table 1. The mean ± SD value of Group 1 (photopolymerizable resin) was 23.16 ± 0.88 μm; Group 2 (composite) was 46.72 ± 2.122 μm; Group 3 (GC pattern) was 23.51 ± 0.736 μm; Group 4 (type III stone) was 19.84 ± 1.017 μm; Group 5 (type IV stone) was 18.72 ± 0.819 μm. 

**Table 1:** Mean values, standard deviation (±SD), minimum and maximum values of Group 1 (photopolymerizable resin), Group 2 (composite), Group 3 ( GC pattern), Group 4 (stone type III), and Group 5 (stone type IV) in µm.

Group	Sample Size	Mean ± SD	Minimum – Maximum
Group 1: Photopolymerizable resin	5	23.16 ± 0.88	22.34 – 24.57
Group 2: Composite	5	46.72 ± 2.122	44.74 – 49.16
Group 3: GC pattern	5	23.51 ± 0.736	22.48 – 24.32
Group 4: Stone III	5	19.84 ± 1.017	18.85 – 21.23
Group 5: Stone IV	5	18.72 ± 0.819	17.57 – 19.59

**Figure 7:**
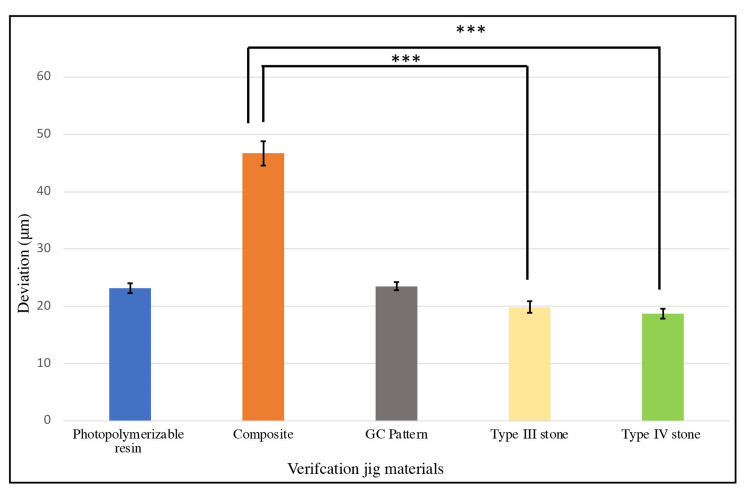
Mean (± SD) 3D deviation values for each verification jig group. Statistical significance differences are indicated by asterisks (Kruskal-Wallis test, p < 0.001).

Type IV stone (Group 5) showed the lowest deviation value by 3D assessment, followed by type III stone (Group 4), Triad (Group 1), GC pattern (Group 3), and composite (Group 2), which showed the highest deviation value (Figure 7). 

The Kruskal-Wallis test indicated statistically significant differences between Groups 2 (composite), 4 (type III stone), and 5 (type IV stone) (p< 0.001). However, there were no significant differences between the other groups. These results suggest low accuracy of composite verification jigs causing significant 3D implant deviation in the produced casts compared to type III and IV stone groups which may affect the clinical outcomes when composite verification jig is used. Photopolymerizable resin and GC pattern groups displayed comparable accuracy with type III and IV stone groups. 

## Discussion

To our knowledge, only one study was conducted to compare the accuracy of different verification jig materials and there is not enough data to determine which is the best accurate material to use for verification jig. Papaspyridakos et al. compared three different materials: GC pattern resin, Fixpeed resin, and Triad gel which are frequently used for making verification jigs. Their results showed no significant differences between these materials when tissue-level parallel implants were used [16]. However, other available materials that can be used for making verification jigs have not been investigated yet. In this study, the accuracy of different verification jig materials was investigated by 3D assessment of the generated fabricated casts constructed from the verification jigs. 

Fabrication of full arch implant-supported fixed prostheses necessitates accurate working casts; the implant position in the cast must be the same as in the mouth to produce an accurate framework [2]. Any discrepancy in the cast will lead to a non-passive prosthesis, which may cause unfavorable strain on the implants and prosthetic components [6,7,22]. 

Numerous factors influence the accuracy of the implant master cast and final implant-prosthesis fits, such as impression technique, impression material, cast material, the skills and experience of the clinician and technician, framework/prosthesis material, and fabrication technique [23-31]. A systematic review by Lee et al. has shown that there was no difference between open-tray and closed-tray impression techniques with three or fewer implants. However, with four or more implants, open-tray impressions were more accurate in producing precise implant master casts than closed-tray impressions [26]. Another study by Vigolo et al. showed that a splinted open-tray impression technique with an acrylic resin splint produced more accurate casts than a non-splinted technique using polyether material [17]. 

Using a verification jig is an important step to determine if there is any discrepancy before fabricating the final framework/prosthesis. Moreover, a retrospective study by Ercoli et al. has shown that all frameworks fit passively in all cases when verification jigs were used. However, most frameworks did not achieve a passive fit when verification jigs were not used [31].

Five different verification jig materials were investigated in this study to determine which material produces the most accurate cast. Light-cure resin and GC pattern are common materials used by dentists to fabricate verification jigs. Composite has also been advocated as a novel material for use in verification jig fabrication. All acrylic resin materials (photopolymerizable resin, GC pattern, and composite) exhibit some polymerization shrinkage, which may impact the accuracy of the verification jigs. To minimize the effect of polymerization shrinkage, all verification jigs were sectioned in three places, separating the jig into six sections. Minimal amounts of material were applied in the sectioned gaps. Thus, only dimensional changes of the additional material connecting the sectioned parts of the jig will affect jig accuracy. Additionally, dental stone has been proposed as an accurate method of verifying the position accuracy of implant analogs in the implant master cast. It is considered one of the most accurate materials for verification jigs due to its low levels of dimensional changes, which are total distortions of 0.08% to 0.2% [21].

The 3D analysis showed that the test casts formed from type IV stone verification jigs more precisely duplicated the exact position of the implant analogs than those constructed of acrylic resin or composite. Also, type III stone demonstrated greater accuracy compared with acrylic resin and composite, but it was not superior to type IV stone. The acrylic resins Triad and GC pattern showed acceptable results, but the composite did not.

The clinical implication that can be concluded from this in vitro study is that all materials used in the study except composite showed promising results with minimum distortion in the produced casts indicating that these materials could be used to create verification jigs. Additionally, type III and IV stone verification jigs exhibited excellent results and using them as a verification jig may improve the framework's passive fit. 

One limitation of this study is that it was conducted in vitro, which differs from clinical situations. The difference in the accuracy of different materials used to fabricate verification jigs in this study may or may not be clinically significant. Jemt defined passive fit as a level that did not cause any long-term clinical consequences, and misfits less than 150 μm were considered acceptable [10]. Other studies suggested that clinically acceptable fits of implant-supported full-arch prostheses vary from 59 to 72 μm and from 91 to 111 μm [10,16]. Therefore, an in vivo comparative study of different materials is required to assess the level of a clinically tolerable misfit. 

Additionally, in our study, the implants were parallel to each other, which is an ideal scenario and different from most clinical cases. It is advisable to conduct a study with tilted implants to determine if the angulation affects the accuracy of the casts produced by the verification jigs.

With advancements in digital technology, computer-aided design/computer-aided manufacturing (CAD/CAM)-milled verification jigs were proposed as an efficient way to confirm the accuracy of the implant's position [32]. It would be interesting to include them in a future study to compare the accuracy of digitally manufactured verification jigs with those fabricated from commonly used materials. 

## Conclusions

Full-arch rehabilitation is a complex restorative procedure influenced by many factors. The passive fit of an implant-supported prosthesis is a critical factor in its success and durability. The present in vitro study concluded that the best verification-jig material for producing the most accurate cast with the best 3D accuracy was type IV stone, followed by type III stone, Triad, GC pattern, and lastly, composite. There were statistically significant differences between Groups 4 (type III stone), 5 (type IV stone), and 2 (composite). Under the limitations of this research, the use of a low-distortion material is recommended for the fabrication of verification jigs for implant-supported fixed dental prostheses to achieve precise results and a passive framework and prosthesis. This will positively impact the quality of the clinical outcome and minimize biological and mechanical complications. Future research to test these materials clinically and assess a tolerable level of prosthesis misfit is recommended.
